# Diagnosing dehydration in the nursing home: international consensus based on a modified Delphi study

**DOI:** 10.1007/s41999-020-00304-3

**Published:** 2020-03-13

**Authors:** Simone J. C. Paulis, Irma H. J. Everink, Ruud J. G. Halfens, Christa Lohrmann, Regina Roller Wirnsberger, Adam L. Gordon, Jos M. G. A. Schols

**Affiliations:** 1 Department of Health Services Research and Care and Public Health Research Institute (CAPHRI), Maastricht University, P.O. Box 616, 6200 MD Maastricht, The Netherlands; 2 Institute of Nursing Science, Medical University of Graz, Graz, Austria; 3 Department of Internal Medicine, Medical University of Graz, Graz, Austria; 4 Division of Medical Sciences and Graduate Entry Medicine, University of Nottingham, Derby, UK; 5 Department of Family Medicine and Care and Public Health Research Institute (CAPHRI), Maastricht University, Maastricht, The Netherlands

**Keywords:** Expert opinion, Diagnostic strategy, Anamnesis, Physical symptoms, Blood tests

## Abstract

**Purpose:**

Even though dehydration is a big problem among nursing home residents, a universally agreed method to diagnose dehydration among nursing home residents is missing. Therefore, this study aimed to establish consensus on a method to diagnose dehydration in this population.

**Methods:**

Using an international Delphi study, 53 experts (physicians and advanced nurse practitioners) were asked to judge various methods to diagnose dehydration on relevance and feasibility in the nursing home. Based on the methods that gained consensus in the first and second round (≥ 75% consensus), a step-by-step diagnostic strategy was developed which was presented to, and judged by, the experts in round three.

**Results:**

After the first and second round, consensus was reached on nine anamnestic items, eight physical symptoms and three blood tests. In the third round, 24 experts agreed with the developed step-by-step diagnostic strategy as a standard to diagnose dehydration in nursing home residents.

**Conclusion:**

This is the first study reaching international consensus on a strategy to diagnose dehydration in the nursing home. This strategy comprehends a presumption phase, where anamnestic items and physical symptoms are examined, followed by a confirmation phase with blood tests to confirm the diagnosis of dehydration. Using this strategy, it is important to take the individual characteristics (e.g. co-morbidity) of the resident and its care environment (e.g. ambient temperature) into account.

**Electronic supplementary material:**

The online version of this article (10.1007/s41999-020-00304-3) contains supplementary material, which is available to authorized users.

## Key summary points

**Aim:**

To assess which method (or combination of methods) are relevant and feasible to diagnose dehydration in nursing home residents.

**Findings:**

International experts agreed on the relevance and feasibility of 9 anamnestic items, 8 physical symptoms and 3 blood tests to diagnose dehydration. This resulted in a diagnostic strategy consisting of a suspicion phase (including anamnestic items and physical symptoms) and a confirmation phase (including blood tests).

**Message:**

This is the first study reaching international consensus about a strategy to diagnose dehydration in the nursing home.

**Electronic supplementary material:**

The online version of this article (10.1007/s41999-020-00304-3) contains supplementary material, which is available to authorized users.

## Introduction

Dehydration is a condition that arises from excessive loss of body water with or without sodium and is a complex care problem, with adverse effects on health and wellbeing [1, 2]. Failure to identify and treat dehydration is associated with reduced quality of life and increased mortality [3–5]. Dehydration often leads to hospital admissions with associated high health care costs [4].

Dehydration often occurs in frail patient populations, such as nursing home residents. Research show widely varying prevalence of dehydration in nursing home residents, ranging from 0.8 to 38.8% depending on the methods used to diagnose dehydration [6]. Health care providers use multiple methods to diagnose dehydration, including physical symptoms (e.g. dry mucous membranes), blood tests (e.g. serum osmolality) and urine tests (e.g. urine specific gravity). However, it is not clear which method, or combination of methods, is the best and most feasible way to diagnose dehydration in nursing home residents [6].

One reason why it is particularly difficult to diagnose dehydration in this target group in a uniform way is that some clinical signs associated with dehydration can also be caused by other conditions common in older adults. For instance, symptoms like tongue furrows, dry mucous membranes, and measurements like urine specific gravity, can be indicative of dehydration but can also be influenced by medications [7, 8]. Another example is the Blood Urea Nitrogen Serum Creatinine Ratio (BUN/S_cr_) which may point to dehydration but also to conditions like renal or heart failure, both common in nursing home residents [9].

A further challenge to diagnose dehydration in nursing home residents is that it is not feasible to use some relevant diagnostic methods in nursing homes in every country [10, 11]. For example, some laboratory tests cannot be taken and/or analyzed in the nursing home itself and the involvement of hospital laboratories is required. This can be time-consuming and lead to delays in results. This in turn delays commencement of treatment resulting in deterioration in residents’ health and avoidable hospital admissions [8, 10, 12].

These factors challenge the adequate and timely detection of dehydration. A universally agreed approach to diagnose dehydration, which is feasible in nursing homes, is needed. The objective of this study is to reach a consensus on a relevant and feasible method (or combination of methods) to diagnose dehydration in nursing home residents by means of a Delphi study.

## Methods

### Research design

To gain consensus on the most relevant and feasible method, or combination of methods, to diagnose dehydration in nursing home residents, a Delphi study was conducted. Three structured rounds of questionnaires were completed. The study was approved by the Medical Ethics Committee of University Hospital Maastricht (2018–0728).

### Data collection and data analysis

**Fig. 1:**
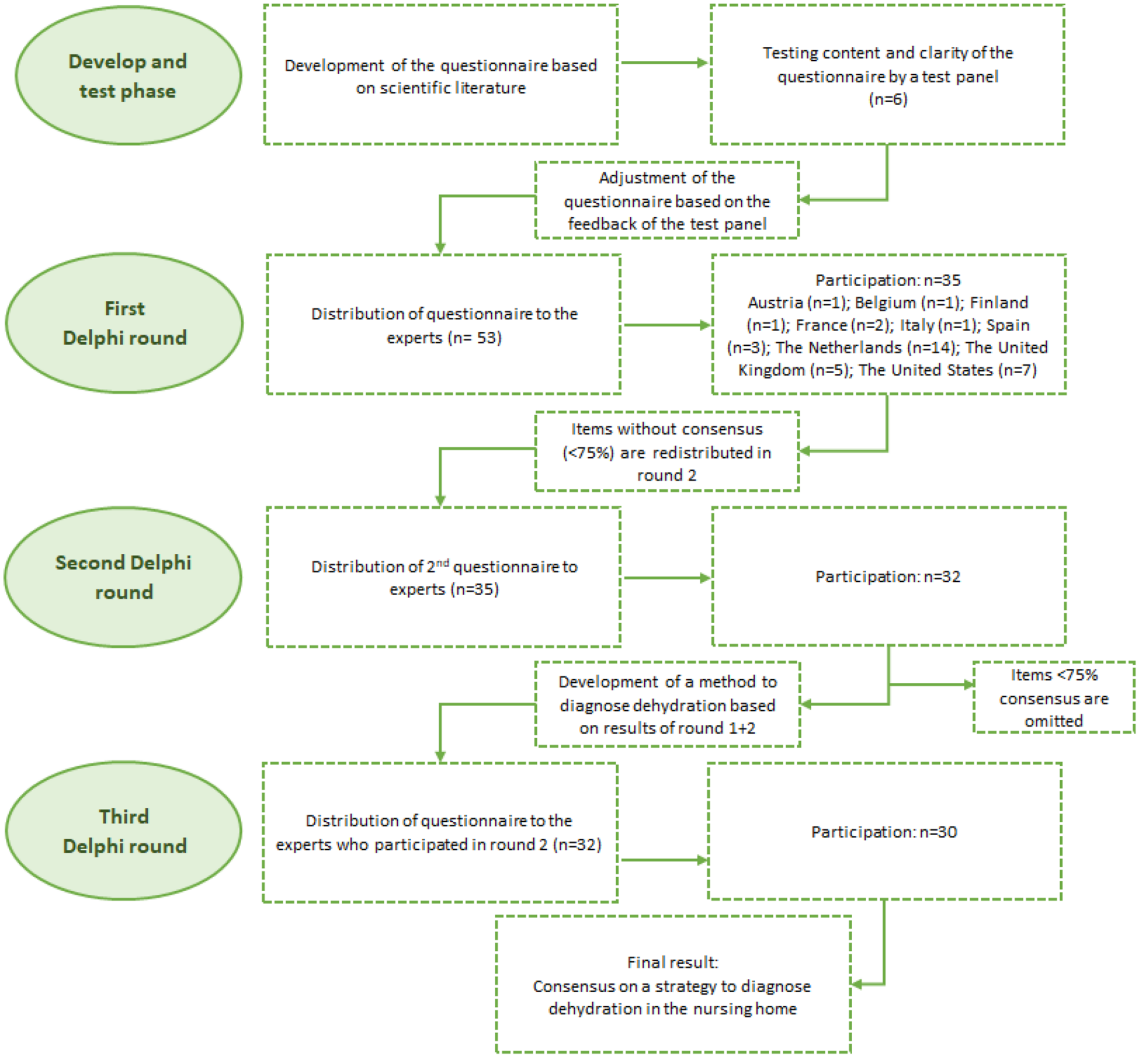
Phases of data collection used in Delphi study

The study consisted of four phases, described in detail below (see Fig. 1).

#### Phase 1: develop and test phase

The questionnaire was based on a systematic review which includes a comprehensive overview of recent literature about dehydration in nursing home residents [6]. The project team critically discussed diagnostic approaches retrieved from the literature and developed a first draft of the questionnaire. To pilot the questionnaire for content and clarity, an international test panel (*n* = 6) obtained from international contacts from the research team, was asked to critically reflect on the draft questionnaire. This international test panel (including panel members from the United Kingdom, The Netherlands and Austria) consisted of an elderly care physician, three geriatricians, a nurse practitioner and an internist. The panel received the questionnaire by e-mail, accompanied by questions about the completeness, structure and clarity of the questionnaire. After critical review by the test panel the questionnaire was adjusted by removing some methods as well as processing several textual changes (see Appendix 1 in Electronic Supplementary Material).

The final questionnaire comprised a general part, including five questions about background characteristics of the participating experts, and four sections representing different methods to diagnose dehydration comprising: anamnestic data (11 items); physical symptoms (10 items); blood tests (5 items); and urine tests (1 item). Experts were asked to indicate whether they thought a method was: (1) a relevant indicator to diagnose dehydration among nursing home residents (yes/no); and (2) feasible for the diagnosis of dehydration in nursing homes (yes/no). After each section, experts were given the opportunity to provide comments or explain their answers. In addition, experts were asked to suggest additional methods to diagnose dehydration, which were not yet included in the questionnaire. Additional methods which were mentioned by > 10% of the experts were included in the Delphi process [13].

##### Selection of experts

Both national and international experts were invited to participate in the Delphi study. Experts were eligible for participation if:

- They were a physician (e.g. general practitioner, geriatrician or elderly care physician [14]), or advanced nurse practitioner, and
- Were currently working with nursing home residents;

Experts were invited to participate from the research team’s professional networks from Austria, Belgium, France, Italy, Norway, Spain, the Netherlands, the United Kingdom and the United States. Not every country has advanced nurse practitioners in their care system. No stratification criteria were used regarding the distribution of experts (physicians vs. advanced nurse practitioners) by country.

The literature suggests a sample size between 30 to 40 experts for a Delphi study [15]. To account for dropout and non-response, 53 experts were invited to participate. Answers from experts who dropped-out during the Delphi rounds were included in the data analysis.

#### Phase 2: first Delphi round

All experts (*n* = 53) who initially agreed to participate in the Delphi study were sent an email on November 26, 2018 through the online Qualtrics software [16]. The email contained information on the aim and content of the Delphi method as well as instructions on questionnaire completion. After two weeks a reminder was sent to experts who had not yet responded.

Experts were asked to indicate whether or not they judged a method to be relevant to the diagnosis of dehydration in nursing home residents. A separate question asked if the method was feasible to conduct in nursing homes. Experts were also given the opportunity to describe methods they deemed important to diagnose dehydration, which were not yet included in the questionnaire. If  > 10% of the experts mentioned the same additional method, it was included in the second round [13].

##### Level of consensus

Based on other studies with a similar Delphi methodology, consensus on a method was reached if ≥ 75% of the experts gave the same answer (yes/no) [17].

#### Phase 3: second Delphi round

A second Delphi round was conducted to seek further consensus (≥ 75% agreement) on methods for which no consensus (< 75% agreement) was reached in the first round. The second questionnaire was sent on February 28, 2019 (see Appendix 1 in Electronic Supplementary Material). A reminder was sent after two weeks to participants who had not yet responded. In this questionnaire, participants received feedback on the methods on which consensus had been reached. Also, all methods for which no consensus was reached in the first round in terms of relevance or feasibility were presented again. Experts were asked to reassess their answers given in the first round by answering ’yes’ or ’no’. To have experts reconsider their initial answers and to seek further consensus, every table contained the distribution of answers provided by experts in the first round together with the individual answers of the expert.

Experts were asked to also assess the relevance and feasibility of additional methods mentioned by > 10% of experts in the first round.

**Fig. 2:**
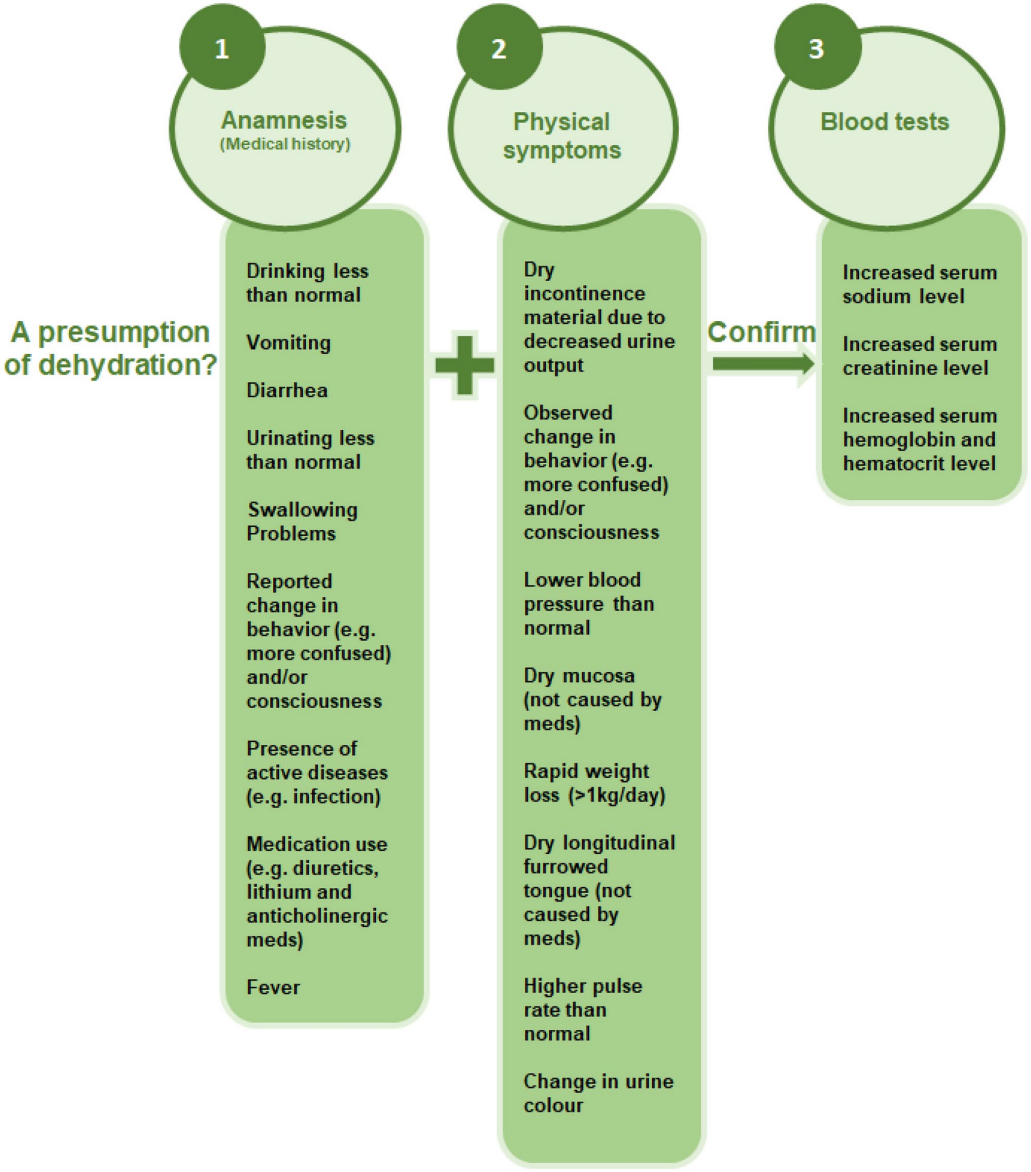
Step-by-step strategy to diagnose dehydration in nursing home residents in the nursing home itself. This strategy can be regarded as a method in which health care professionals first have to pay attention to the items in the presumption phase, where after the diagnosis of dehydration is established in the confirmation phase taking into account the individual characteristics of a resident and the characteristics of the care environment

Methods which had failed to reach consensus after the second round were excluded. The reason for this was that after giving the same answer in two consecutive rounds, it was not likely that experts would change their answers in the third round [18, 19]. The methods for which consensus was reached on relevance and feasibility were used to develop a step-by-step diagnostic strategy based on regular clinical practice to diagnose dehydration in nursing home residents (see Fig. 2) [20].

#### Phase 4: third Delphi round

In the third and final round, which started on May 28, 2019 experts were asked to indicate whether or not they agreed with this strategy as a standard to diagnose dehydration in nursing home residents (see Appendix 1 in Electronic Supplementary Material). Experts could answer this question with ’yes’ or ’no’. If the answer was ’no’, experts were asked to explain their answer. A reminder was sent after two weeks to the experts who did not respond yet in the third round.

All results were analyzed using descriptive statistics in the software program SPSS Statistics 25 (IBM) [21].

## Results

### First Delphi round

#### Demographic characteristics

In total, 35 out of 53 invited experts participated in the first Delphi round. Reasons for non-response were not provided. Twenty-two (62.9%) were physicians and 13 (37.1%) advanced nurse practitioners. Participants worked in Austria (*n* = 1), Belgium (*n* = 1), Finland (*n* = 1), France (*n* = 2), Italy (*n* = 1), Spain (*n* = 3), The Netherlands (*n* = 14), the United Kingdom (*n* = 5) and the United States (*n* = 7) (see Fig. 1). The mean years of working experience in providing care for nursing home residents were 18.4 years (SD 8.9). Most experts (68.6%) worked in a nursing home with no formal agreements with a hospital about 24-h laboratory diagnostic support.

#### Consensus after first round

Anamnesis: Of the 11 methods in the section ‘anamnesis’, consensus on relevance and feasibility of a method to diagnose dehydration was reached for 8 (72.7%) methods: ‘drinking less than normal/decreased fluid intake’, ‘presence of active disease(s)’, ‘fever’, ‘vomiting’, ‘diarrhea’, ‘swallowing problems’, ‘reported change in behavior’ and ‘use of medication’.

Physical symptoms: In the section ‘physical symptoms’, consensus was achieved for 3 out of 10 methods (30%). These were ‘lower blood pressure than normal’, ‘dry incontinence material due to decreased urine output’ and ‘observed change in behavior’.

Blood- and urine testing: In the section blood- and urine testing, no consensus could be reached in terms of relevance and feasibility (see Table 1). Experts mentioned some additional methods in the first round. ‘An increased Blood Urea Nitrogen (BUN)’ (*n* = 4) and ‘an increased serum urea’ (*n* = 6) were carried forward to the second round as they were mentioned by > 10% of the experts.

**Table 1:** Methods consensus round 1 and 2

	Relevant to diagnose dehydration among nursing home residents (yes) Round 1 (*n* = 35)	Feasible to diagnose dehydration in the nursing home (yes) Round 1 (*n* = 35)	Relevant to diagnose dehydration among nursing home residents (yes) Round 2 (*n* = 32)	Feasible to diagnose dehydration in the nursing home (yes) Round 2 (*n* = 32)
**Anamnestic data**				
Drinking less than normal/decreased fluid intake	*n* = 34 (97.1%)^a^	*n* = 32 (91.4%)^a^		
Vomiting	*n* = 34 (97.1%)^a^	*n* = 33 (94.3%)^a^		
Diarrhea	*n* = 34 (97.1%)^a^	*n* = 33 (94.3%)^a^		
Urinating less than normal	*n* = 33 (94.3%)^a^	*n* = 23 (65.7%)		*n* = 28 (87.5%)^a^
Swallowing problems	*n* = 32 (91.4%)^a^	*n* = 32 (91.4%)^a^		
Change in behavior (e.g. more confused) and/ or consciousness	*n* = 32 (91.4%)^a^	*n* = 29 (82.9%)^a^		
Presence of active disease(s) (e.g. renal failure, infection, active co-pathology such as DM	*n* = 31 (88.6%)^a^	*n* = 30 (85.7%)^a^		
Use of medication (e.g. diuretic medication, lithium, anticholinergic meds, ACE-inhibitors, beta-blockers)	*n* = 31 (88.6%)^a^	*n* = 28 (80.0%)^a^		
Fever	*n* = 30 (85.7%)^a^	*n* = 31 (88.6%)^a^		
Sweating	*n* = 28 (80.0%)^a^	*n* = 24 (68.6%)		*n* = 20 (62.5%)
Thirst	*n* = 22 (62.9%)	*n* = 15 (42.9%)	*n* = 17 (53.1%)	*n* = 10 (31.3%)
**Physical symptoms**				
Dry incontinence material due to decreased urine output	*n* = 33 (94.3%)^a^	*n* = 29 (82.9%)^a^		
Change in behavior (e.g. more confused) and/or consciousness	*n* = 31 (88.6%)^a^	*n* = 27 (77.1%)^a^		
Lower blood pressure than normal	*n* = 31 (88.6%)^a^	*n* = 29 (82.9%)^a^		
Dry mucosa (not caused by medication)	*n* = 29 (82.9%)^a^	*n* = 26 (74.3%)		*n* = 28 (87.5%)^a^
Rapid weight loss (> 1 kg per day)	*n* = 26 (74.3%)	*n* = 27 (77.1%)^a^	*n* = 28 (87.5%)^a^	
Dry longitudinal furrowed tongue (not caused by medication)	*n* = 25 (71.4%)	*n* = 22 (62.9%)	*n* = 30 (93.8%)^a^	*n* = 28 (87.5%)^a^
Higher pulse rate than normal	*n* = 25 (71.4%)	*n* = 26 (74.3%)	*n* = 29 (90.6%)^a^	*n* = 30 (93.8%)^a^
Change in urine colour	*n* = 25 (71.4%)	*n* = 27 (77.1%)^a^	*n* = 27 (84.4%)^a^	
Hyperthermia	*n* = 21 (60.0%)	*n* = 24 (68.6%)	*n* = 22 (68.8%)	*n* = 27 (84.4%)^a^
Poor skin turgor	*n* = 21 (60.0%)	*n* = 22 (62.9%)	*n* = 19 (59.4%)	*n* = 20 (62.5%)
**Blood tests**				
Increased serum sodium level	*n* = 30 (85.7%)^a^	*n* = 23 (65.7%)		*n* = 27 (84.4%)^a^
Increased serum creatinine level	*n* = 28 (80.0%)^a^	*n* = 25 (71.4%)		*n* = 27 (84.4%)^a^
Increased serum osmolality	*n* = 26 (74.3%)	*n* = 17 (48.6%)	*n* = 30 (93.8%)^a^	*n* = 18 (56.3%)
Higher blood glucose level (in case of diabetes mellitus)	*n* = 23 (65.7%)	*n* = 28 (80.0%)^a^	*n* = 22 (68.8%)	
Increased serum hemoglobin and hematocrit level	*n* = 23 (65.7%)	*n* = 25 (71.4%)	*n* = 25 (78.1%)^a^	*n* = 27 (84.4%)^a^
**Urine tests**				
Increased urine glucose level (in case of Diabetes Mellitus)	*n* = 13 (37.1%)	*n* = 19 (54.3%)	*n* = 5 (15.6%)	*n* = 12 (37.5%)

^a^Consensus reached (≥ 75%)

### Second Delphi round

In total, 32 experts participated in the second round (see Fig. 1). Reasons for drop-out were not provided.

#### Consensus after second round

Anamnesis: Of the anamnestic methods without consensus in the first round, no consensus could be reached in terms of relevance and feasibility on the method ‘thirst’ and ‘sweating’ to diagnose dehydration among nursing home residents. Consensus was reached for ‘urinating less than normal’.

Physical symptoms: Of the remaining seven physical symptoms without consensus after the first Delphi round, consensus was reached for relevance and feasibility on five methods: ‘dry mucosa’, ‘dry longitudinal furrowed tongue’, ‘a higher pulse rate than normal’, ‘rapid weight loss’ and ‘change in urine colour’.

Blood- and urine testing: Out of five methods in the section blood tests, ‘increased serum hemoglobin’ and ‘hematocrit level’, ‘increased serum creatinine level’ and ‘increased serum sodium level’ reached consensus on relevance and feasibility. No consensus could be reached on the methods in the section ‘urine tests’. Increased serum urea and BUN were mentioned as additional methods in the section blood tests in the first Delphi round. More than 75% of the experts assessed increased serum urea as relevant but felt that it was not feasible to test in the nursing home. BUN did not achieve consensus for relevance or feasibility.

### Third Delphi round

Based on the items that reached consensus on relevance and feasibility after the second round, a diagnostic strategy to assess dehydration among nursing home residents was developed. This strategy comprised a presumption phase and a confirmation phase. The presumption phase included the anamnestic items and physical symptoms on which consensus was reached, whilst the confirmation phase included the blood tests on which consensus was reached. This strategy can be regarded as a step-by-step plan whereby health care professionals focus first on the presumption phase before moving on to confirm their diagnosis of dehydration in the confirmation phase (see Fig. 2).

In total, 30 experts participated in the third round (see Fig. 1) seeking agreement on the diagnostic strategy. Twenty-four experts agreed on the strategy as the standard to diagnose dehydration among nursing home residents. Experts who disagreed with the strategy were largely physicians (83%) and worked in the Netherlands (*n* = 3), the United Kingdom (*n* = 2) and Finland (*n* = 1). The main reason they disagreed with the strategy was that the strategy did not specify how many methods/items should be fulfilled to start the confirmation phase.

## Discussion

This study established a structured approach to diagnose dehydration in nursing homes comprising anamnestic items, physical symptoms and blood tests. This approach is supported by professional consensus from healthcare experts across nine countries.

The E-SPEN guideline and a diagnostic accuracy study suggested that serum osmolality was the best test to diagnose dehydration among older adults and nursing home residents [22, 23]. Some elements from the E-SPEN guideline support the results of this Delphi study. The difference is that we focused this Delphi study entirely on nursing home residents and the feasibility of methods in nursing homes. Serum osmolality was not supported by our Delphi exercise when taking feasibility into account, allowing for the lack of laboratory access from nursing homes. In addition, our consensus strategy incorporated several physical signs (e.g. including the dry furrowed tongue, dry mucosa and changes in pulse rate and blood pressure), the importance of which has been previously contested due to their lack of accuracy [23]. Literature suggests that these physical symptoms are individually less useful, but a combination of physical symptoms may identify dehydration [22]. A patient-tailored approach in the nursing home setting seems desirable as indicated by experts in this Delphi study. Our approach here was pragmatic, with an emphasis on feasibility, and the resulting recommendation incorporates these signs in the presumption phase, only establishing the diagnosis with supporting blood tests in the confirmation phase.

In addition, although the strategy developed in our study indicates that if any of the above-mentioned anamnestic items and physical symptoms is present, this could be a reason for further diagnostics (blood testing), prioritization of the items was not done. Four out of six experts in the third Delphi round disagreed with the strategy due to the absence of prioritization. The rationale for excluding prioritization was that the importance of any of these items is highly dependent on the individual characteristics of a resident, as well as other risk factors such as characteristics of the care environment for developing dehydration that might be present [24–26]. This means that for one resident, only one anamnestic item and one physical symptom might trigger further blood testing, while for another resident 3 or 4 items be present before deciding to perform a blood test. Therefore, it is also recommended to take into account the individual characteristics (e.g. co-morbidity) and the resident’s care environment (e.g. ambient temperature) in the decision-making process if further diagnostics (blood tests) are needed [2, 5].

We did not rank items included in the diagnostic strategy in terms or order of importance. It is, though, evident from Table 1 that there was greater consensus around the importance of particular items. ‘Drinking less than normal/decreased fluid intake’, ‘vomiting’, ‘diarrhea’, ‘swallowing problems’, ‘urinating less than normal’, ‘reported change in behaviour’ and ‘a higher pulse rate than normal’ scored the highest level of agreement for both relevance and feasibility. There is a strong emphasis amongst these in detecting a change from normality. This requires detailed knowledge of the resident and emphasises the important of nursing home staff in triggering the presumption phase. The technical challenges of supporting the anamnestic items and physical symptoms in the strategy are recognised, with evidence that decreased oral intake, for example, may be difficult for nursing staff to recognise [27, 28]. More structured approaches to these anamnestic items and physical symptoms included in the strategy are required and should be the focus of future research.

## Strengths and limitations

The strengths of this study rise from a highly structured and objective approach, comprising thorough literature review, followed by structured consensus informed by experts from a number of countries [15]. The international nature of the participants increases the generalisability of our findings. The study had good response rates (varying between 66 and 93.8%) compared with the broader Delphi literature [29, 30].

The results from this Delphi study may be generalizable to other settings, besides the nursing home, in which older adults receive care. The strategy is tailored to the needs of individual patients and takes into account the heterogeneity of older adults. Nevertheless, there may be differences in the feasibility of the methods when moving between different settings. We would recommend further consensus work before these findings are transferred to other settings.

A limitation is the failure to rank methods in terms of importance for the final diagnostic strategy. Whilst this made it easier to achieve consensus, it provides less specific guidance on which items should be prioritized. This was, though, a conscious decision, as inferring strength of association between anamnestic signs and physical symptoms and the likelihood of dehydration based upon consensus alone would be conceptually flawed. The resulting strategy allows healthcare professionals to structure their approach and draws their attention to areas of agreed importance when presuming and confirming a diagnosis of dehydration.

A potential bias in this study is that the majority of the participants came from one specific country (the Netherlands). This might have led to selection bias. However, subanalyses showed that experts from the Netherlands did not indicate more or different methods to be relevant or feasible to diagnose dehydration compared to experts from other countries. An additional strength was that the Delphi study consisted of multiple rounds, between which individual answers with summarized group responses were distributed to each expert. This allowed experts to make a well-considered (re)assessment on the relevance and feasibility of a method [31].

Finally, although this strategy was assessed on both relevance and feasibility, we are aware that formal agreements with a hospital about 24-h laboratory diagnostics is not standard practice in every country. Therefore, when implementing the strategy, clear agreements with hospitals on blood testing should be made.

## Conclusion

This Delphi study produced a strategy to diagnose dehydration using a range of anamnestic items, physical symptoms and blood tests. Research to validate these recommendations is required especially because of the high heterogeneity and multimorbidity in this group. The strategy encompasses a broad range of items. As various items in the presumption phase of the strategy can only be identified by a health care professional who frequently interacts with the resident, effective interdisciplinary working between nursing staff and physicians in this phase is essential. Further research should focus on this collaboration and the barriers and facilitators to this from the perspective of both parties. Additionally, identifying changes in residents in busy routine care practice in the context of multimorbidity, functional dependency and cognitive impairment can be challenging. Research to develop reliable approaches to the anamnestic items and physical symptoms are, therefore, important.

## Electronic supplementary material

Below is the link to the electronic supplementary material.

Supplementary file1 (DOCX 1002 kb)
