# Supplementary material for: Diagnosing dehydration in the nursing home: international consensus based on a modified Delphi study
Source: Eur Geriatr Med. 2020 Mar 13;11(3):393–402. doi: 10.1007/s41999-020-00304-3 (PMC7280358; doi:10.1007/s41999-020-00304-3)
Supplement: Supplementary file 1 — Supplementary file1 (DOCX 1002 kb) [file 41999_2020_304_MOESM1_ESM.docx]

**Title:** Diagnosing dehydration in the nursing home: International consensus based on a modified Delphi study

**Journal name:** European Geriatric Medicine

**Authors:** Simone J.C. Paulis, MSc; Irma H.J. Everink, PhD; Ruud J.G. Halfens, PhD; Christa Lohrmann, PhD; Regina Roller Wirnsberger, MD MME ; Adam L. Gordon, PhD MBChB; Jos M.G.A. Schols, MD, PhD.

**Corresponding author:** Simone J.C. Paulis, MSc, Department of Health Services Research and Care and Public Health Research Institute (CAPHRI), Maastricht University, P.O. Box 616, 6200MD Maastricht, The Netherlands^.^ E-mail: [s.paulis@maastrichtuniversity.nl](mailto:s.paulis@maastrichtuniversity.nl); telephone +31 630421453

Delphi study dehydration

(questionnaire Delphi round 1)

**Welcome!**
Thank you for participating in this Delphi study. This questionnaire consists of four sections:

1. Anamnesis

2. Physical diagnosis

3. Blood tests

4. Urine tests

**Background information**

Your responses will be treated confidentially. We only ask for your name to be able to link the different rounds of questionnaires to each other. Further background information will be used for demographic analyses only.

Q1 **Name:**

___________________________________________________________

Q2 **Country where you work:** ____________________________________________________________

Q3 **Profession:**

- Physician (1)
- Advanced Nurse Practitioner (2)

Q4 **Years of experience in providing care for older people**

________________________________________________________________

Q5 **Are you currently working with nursing home residents?**

- Yes (1)
- No (2)

Q6 **Is the nursing home you are regularly attending, located in the proximity (within 1 mile/1.6 kilometer) of a hospital?**

- Yes (1)
- No (2)

Q7 **Does the nursing home you are regularly attending have formal agreements with a hospital about 24 hr laboratory diagnostics?**

- Yes (1)
- No (2)

**Section 1: Anamnesis**

Q8 **Please indicate which anamnestic items you believe are relevant to diagnose dehydration in nursing home residents. Please also indicate which of the items you believe are feasible to diagnose dehydration in a nursing home itself.**

|  | Relevant to diagnose dehydration among nursing home residents | | Feasible to diagnose dehydration in the nursing home | |
| --- | --- | --- | --- | --- |
|  | Yes (1) | No (2) | Yes (1) | No (2) |
| Drinking less than normal / decreased fluid intake (1)  Thirst (2)  Presence of active disease(s) (e.g. renal failure, infection, active co-pathology such as diabetes mellitus) (3)  Fever (4)  Sweating (5)  Vomiting (6)  Diarrhea (7)  Swallowing problems (8)  Change in behaviour (e.g.more confused) and / or onsciousness (9)  Urinating less than normal (10)  Use of medication (e.g. diuretic medication, lithium, anticholinergic medication, ACE-inhibitors, beta-blockers) (11) |  |  |  |  |

Q9 **Are there any anamnestic items missing (please mention which other item(s) you consider important)?**

|  | Relevant to diagnose dehydration among nursing home residents | | Feasible to diagnose dehydration in the nursing home | |
| --- | --- | --- | --- | --- |
|  | Yes (1) | No (2) | Yes (1) | No (2) |
| Other (1) |  |  |  |  |
| Other (2) |  |  |  |  |
| Other (3) |  |  |  |  |

Q10 **Which anamnestic items from the lists above do you consider important when diagnosing chronic dehydration?**

________________________________________________________________

________________________________________________________________

Q11 **If you want to motivate or explain your answers please provide these additional comments below.**

________________________________________________________________

________________________________________________________________

**Section 2: Physical diagnosis**

Q12 **Please indicate which physical symptom(s) you believe are relevant to diagnose dehydration in nursing home residents. Please also indicate which physical symptom(s) you believe are feasible to diagnose dehydration in a nursing home itself.**

|  | Relevant to diagnose dehydration among nursing home residents | | Feasible to diagnose dehydration in the nursing home | |
| --- | --- | --- | --- | --- |
|  | Yes (1) | No (2) | Yes (1) | No (2) |
| Dry mucosa (not caused by medication) (1)  Dry longitudinal furrowed tongue (not caused by medication) (2)  Hyperthermia (3)  Poor skin turgor: if yes, please describe in the box where to pitch the skin turgor (4)  Higher pulse rate than normal (5)  Lower blood pressure than normal (6)  Rapid weight loss (>1 kg per day) (7)  Dry incontinence material due to decreased urine output (8)  Change in urine colour (9)  Change in behavior (e.g. more confused) and / or consciousness (10) |  |  |  |  |

Q13 **Are there any physical symptoms missing (please mention which other item(s) you consider important)?**

|  | Relevant to diagnose dehydration among nursing home residents | | Feasible to diagnose dehydration in the nursing home | |
| --- | --- | --- | --- | --- |
|  | Yes (1) | No (2) | Yes (1) | No (2) |
| Other (1) |  |  |  |  |
| Other (2) |  |  |  |  |
| Other (3) |  |  |  |  |

Q14 **If you want to motivate or explain your answers please provide these additional comments below.**

________________________________________________________________

________________________________________________________________

**Section 3: Blood testing**

Q15 **Please indicate which blood test(s) you believe are relevant to diagnose dehydration in nursing home residents. Please also indicate which blood test(s) you believe are feasible to diagnose dehydration in a nursing home itself.**

|  | Relevant to diagnose dehydration among nursing home residents | | Feasible to diagnose dehydration in the nursing home | |
| --- | --- | --- | --- | --- |
|  | Yes (1) | No (2) | Yes (1) | No (2) |
| Higher blood glucose level (in case of Diabetes Mellitus) (1) |  |  |  |  |
| Increased serum hemoglobin and hematocrit level (2) |  |  |  |  |
| Increased serum creatinine level (3) |  |  |  |  |
| Increased serum osmolality (4) |  |  |  |  |
| Increased serum sodium level (5) |  |  |  |  |

Q16 **Are there any blood tests missing (please mention which other item(s) you consider important)?**

|  | Relevant to diagnose dehydration among nursing home residents | | Feasible to diagnose dehydration in the nursing home | |
| --- | --- | --- | --- | --- |
|  | Yes (1) | No (2) | Yes (1) | No (2) |
| Other (1) |  |  |  |  |
| Other (2) |  |  |  |  |
| Other (3) |  |  |  |  |

Q17 **If you want to motivate or explain your answers please provide these additional comments below.**

________________________________________________________________

________________________________________________________________

**Section 4: Urine testing**Q18 **Please indicate which urine test(s) you believe are relevant to diagnose dehydration in nursing home residents. Please also indicate and which urine test(s) you believe are feasible to diagnose dehydration in a nursing home itself.**

|  | Relevant to diagnose dehydration among nursing home residents | | Feasible to diagnose dehydration in the nursing home | |
| --- | --- | --- | --- | --- |
|  | Yes (1) | No (2) | Yes (1) | No (2) |
| Increased urine glucose level (in case of Diabetes Mellitus) (1) |  |  |  |  |

Q19 **Are there any urine tests missing (please mention which other item(s) you consider important)?**

|  | Relevant to diagnose dehydration among nursing home residents | | Feasible to diagnose dehydration in the nursing home | |
| --- | --- | --- | --- | --- |
|  | Yes (1) | No (2) | Yes (1) | No (2) |
| Other (1) |  |  |  |  |
| Other (2) |  |  |  |  |
| Other (3) |  |  |  |  |

Q20 **If you want to motivate or explain your answers please provide these additional comments below.**

________________________________________________________________

________________________________________________________________

**Thank you for your valuable cooperation in this Delphi round! Finish this questionnaire and record your answers by clicking on the ´next´ button.**

Delphi study dehydration

(questionnaire Delphi round 2)

**Welcome!**
Thank you for participating in the second round of this Delphi study. This questionnaire consists of 4 sections:

1. Anamnesis

2. Physical diagnosis

3. Blood tests

4. Urine tests

In every section the results from the first Delphi round will be presented to you. The items on which consensus (≥75%) was reached, are displayed in graphics.

Furthermore, in every section you will find a table where the items are presented on which consensus was not reached in terms of relevance or feasibility. These items will be presented to you again. First, the distribution of answers provided by participants in the first round are given, accompanied by the answer you gave in the first round. We kindly ask you to reassess your answer to this item by answering ''yes'' or ''no''.

Additional items mentioned by >10% of the participants are now also included as potential methods to diagnose dehydration. This was ontly the case for items in the section ''blood testing''. Please also assess the relevance and feasibility of these additional items by answering ''yes'' or ''no''.

*Please read every question carefully whether it concerns **relevance** or **feasibility**.

**Anamnesis**Consensus (≥75%) was reached on the following anamnestic items to diagnose dehydration among nursing home residents in terms of relevance


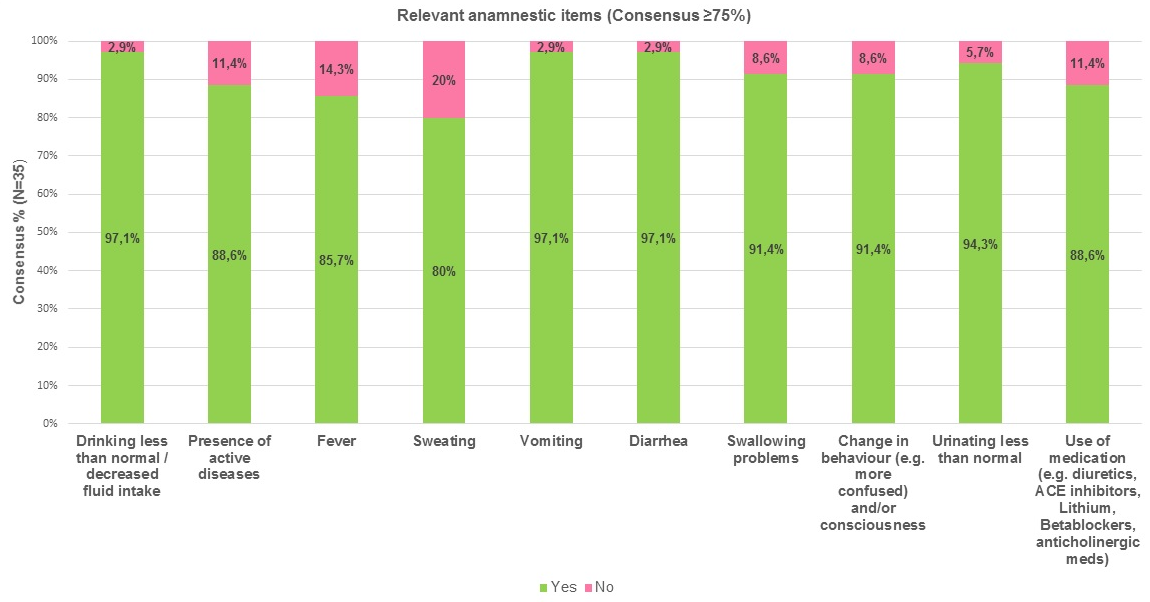


No consensus was reached on the following anamnestic items in terms of relevance to diagnose dehydration among nursing home residents

Distribution of answers in round 1 Your answer in round 1

Thirst Yes: 62.9%
 No: 37.1%

Q1 **We ask you to reassess your initial answer. Please press ''yes'' or ''no''. Is the following anamnestic item relevant to diagnose dehydration among nursing home residents?
 
Thirst**

- Yes (1)
- No (2)

**Anamnesis**Consensus (≥75%) was reached on the following anamnestic items to diagnose dehydration in the nursing home in terms of feasibility


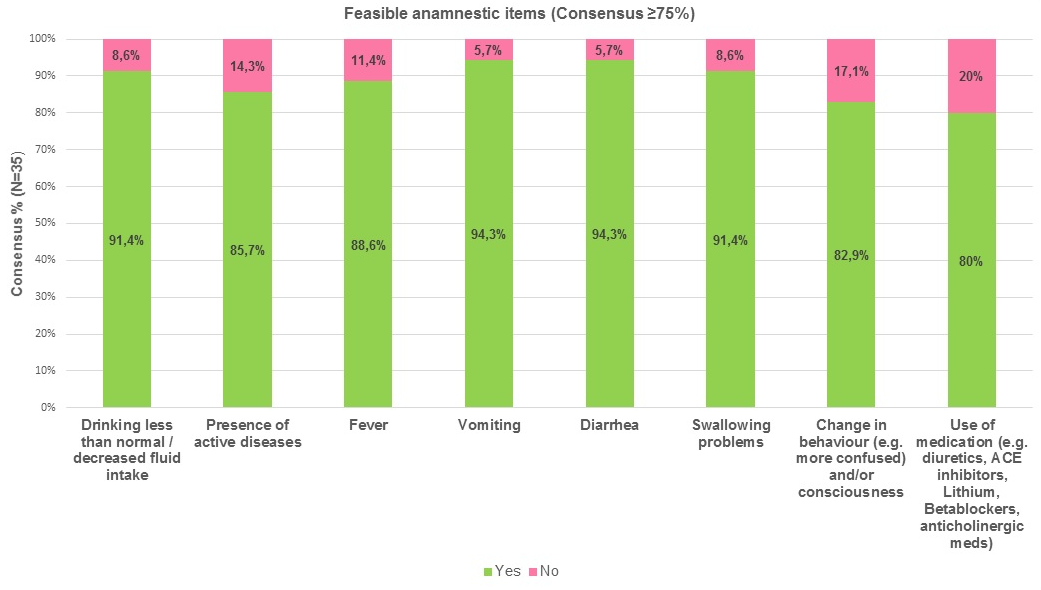


No consensus could be reached on the following anamnestic items in terms of feasibility to diagnose dehydration in the nursing home:

Distribution of answers in round 1 Your answer in round 1

Thirst Yes: 42.9%
 No: 57.1%

Sweating Yes: 68.6%
 No: 31.4%

Urinating less Yes: 65.7%

than normal No: 34.3%

Q2 **We ask you to reassess your initial answer. Please press ''yes'' or ''no''. Are the following anamnestic items feasible to diagnose dehydration in the nursing home?
 
Thirst**

- Yes (1)
- No (2)

**Sweating**

- Yes (1)
- No (2)

**Urinating less than normal**

- Yes (1)
- No (2)

**Physical diagnosis**Consensus (≥75%) was reached on the following physical symptoms to diagnose dehydration among nursing home residents in terms of relevance


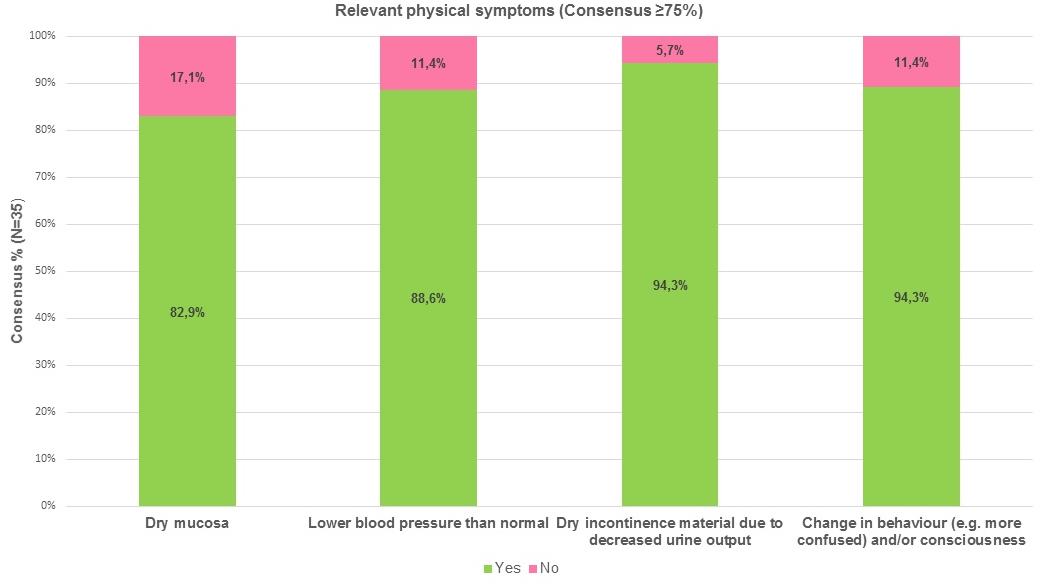


No consensus was reached on the following physical symptoms in terms of relevance to diagnose dehydration among nursing home residents

Distribution of answers in round 1 Your answer in round 1
Dry longitudinal furrowed Yes: 71.4%

tongue (not caused No: 28.6

by medication)

Hyperthermia Yes: 60%
 No: 40%

Poor skin turgor Yes: 60%
 No: 40%

Higher pulse rate Yes: 71.4%

than normal No: 28.6%

Rapid weight loss Yes: 74.3%

(>1kg per day) No: 25.7%

Change in urine Yes: 71.4%

Colour No: 28.6%

Q3 **We ask you to reassess your initial answer. Please press ''yes'' or ''no''. Are the following physical symptoms relevant to diagnose dehydration among nursing home residents?**
**Dry longitudinal furrowed tongue (not caused by medication)**

- Yes (1)
- No (2)

**Hyperthermia**

- Yes (1)
- No (2)

**Poor skin turgor**

- Yes (1)
- No (2)

**Higher pulse rate than normal**

- Yes (1)
- No (2)

**Rapid weight loss (>1kg per day)**

- Yes (1)
- No (2)

**Change in urine colour**

- Yes (1)
- No (2)

**Physical diagnosis**Consensus (≥75%) was reached on the following physical symptoms to diagnose dehydration in the nursing home in terms of feasibility


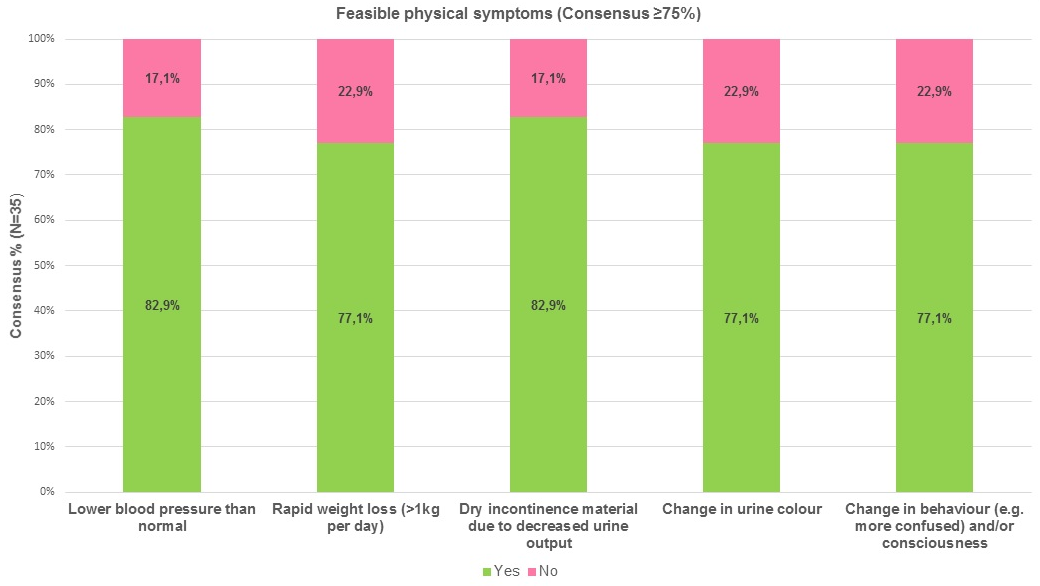


No consensus was reached on the following physical symptoms in terms of feasibility to diagnose dehydration in the nursing home

Distribution of answers in round 1 Your answer in round 1

Dry mucosa (not caused Yes: 74.3%

by medication) No: 25.7%  
Dry longitudinal furrowed Yes: 62.9%

tongue (not caused by No: 37.1%

medication)

Hyperthermia Yes: 68.6%
 No: 31.4%  
Poor skin turgor Yes: 62.9%
 No: 37.1%

Higher pulse rate than Yes: 74.3%

Normal No: 25.7%

Q4 **We ask you to reassess your initial answer. Please press ''yes'' or ''no''. Are the following physical symptoms feasible to diagnose dehydration in the nursing home?**

**Dry mucosa (not caused by medication)**

- Yes (1)
- No (2)

**Dry longitudinal furrowed tongue (not caused by medication)**

- Yes (1)
- No (2)

**Hyperthermia**

- Yes (1)
- No (2)

**Poor skin turgor**

- Yes (1)
- No (2)

**Higher pulse rate than normal**

- Yes (1)
- No (2)

**Blood testing**Consensus (≥75%) was reached on the following blood tests to diagnose dehydration among nursing home residents in terms of relevance


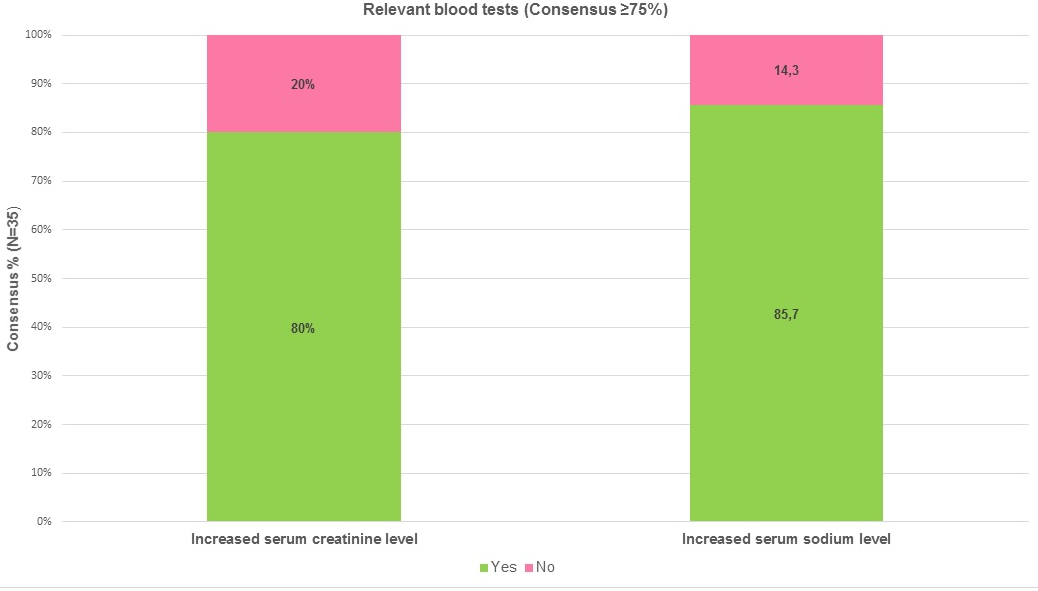


No consensus was reached on the following blood tests in terms of relevance to diagnose dehydration among nursing home residents

Distribution of answers in round 1 Your answer in round 1

Higher blood glucose level

(in case of DM) Yes: 65.7%

No: 34.3%

Increased serum Yes: 65.7%

hemoglobin and No: 34.3%

hematocrit level

Increased serum Yes: 74.3%

Osmolality No: 25.7%

Q5 **We ask you to reassess your initial answer. Please press ''yes'' or ''no''. Are the following blood tests relevant to diagnose dehydration among nursing home residents?**

**Higher blood glucose level (in case of DM)**

- Yes (1)
- No (2)

**Increased serum hemoglobin and hematocrit level**

- Yes (1)
- No (2)

**Increased serum osmolality**

- Yes (1)
- No (2)

**Blood testing**Consensus (≥75%) was reached on the following blood tests to diagnose dehydration in the nursing home in terms of feasibility


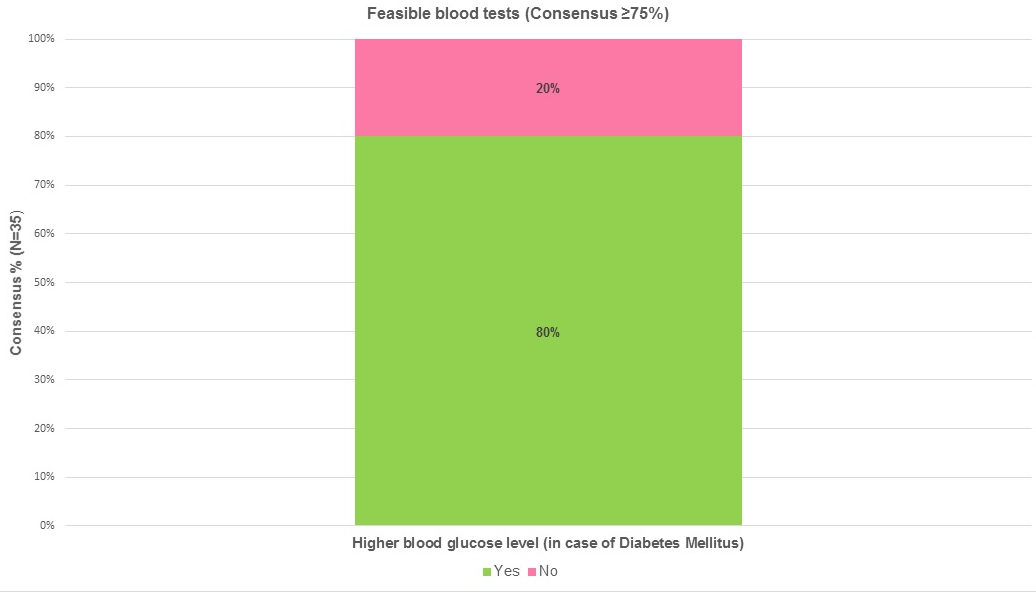


No consensus was reached on the following blood tests in terms of feasibility to diagnose dehydration in the nursing home

Distribution of answers in round 1 Your answer in round 1

Increased serum hemoglobin Yes: 71.4%

and hematocrit level No: 28.6%

Increased serum creatinine Yes: 71.4%

Level No: 28.6%

Increased serum osmolality Yes: 48.6%

No: 51.4%

Increased serum sodium level Yes: 65.7%

No: 34.4%

Q6 **We ask you to reassess your initial answer. Please press ''yes'' or ''no''. Are the following blood tests feasible to diagnose dehydration in the nursing home?**

**Increased serum hemoglobin and hematocrit level**

- Yes (1)
- No (2)

**Increased serum creatinine level**

- Yes (1)
- No (2)

**Increased serum osmolality**

- Yes (1)
- No (2)

**Increased serum sodium level**

- Yes (1)
- No (2)

**Blood testing**Q7 Based on additional suggestions provided by participants in the first round, the following blood tests are added as potential methods to diagnose dehydration. Please indicate whether or not you think these blood tests are relevant to diagnose dehydration among nursing home residents by pressing ''yes'' or ''no''.

**Increased Blood Urea Nitrogen (BUN)**

- Yes (1)
- No (2)

**Increased serum Urea**

- Yes (1)
- No (2)

**Blood testing**Q8 Based on additional suggestions provided by participants in the first round, the following blood tests are added as potential methods to diagnose dehydration. Please indicate whether or not you think these blood tests are feasible to diagnose dehydration among nursing home residents by pressing ''yes'' or ''no''.

**Increased Blood Urea Nitrogen (BUN)**

- Yes (1)
- No (2)

**Increased serum Urea**

- Yes (1)
- No (2)

**Urine testing**No consensus was reached on urine tests to diagnose dehydration among nursing home residents in terms of relevance

No consensus was reached on the following urine test in terms of relevance to diagnose dehydration among nursing home residents

Distribution of answers in round 1 Your answer in round 1

Increased urine glucose level Yes: 37.1%

(in case of DM) No: 62.9%

Q9 **We ask you to reassess your initial answer. Please press ''yes'' or ''no''. Is the following urine test relevant to diagnose dehydration among nursing home residents?**

**Increased urine glucose level (in case of DM)**

- Yes (1)
- No (2)

**Urine testing**

No consensus was reached on urine tests to diagnose dehydration in the nursing home in terms of feasibility

No consensus was reached on the following urine test in terms of feasibility to diagnose dehydration in the nursing home

Distribution of answers in round 1 Your answer in round 1

Increased urine glucose level Yes: 54.3%

(in case of DM) No: 45.7%

Q10 **We ask you to reassess your initial answer. Please press ''yes'' or ''no''. Is the following urine test feasible to diagnose dehydration in the nursing home?**

**Increased urine glucose level (in case of DM)**

- Yes (1)
- No (2)

**Thank you for your valuable cooperation!**

Delphi study dehydration

(questionnaire Delphi round 3)

**Welcome!**

Thank you for participating in the third and final round of this Delphi study knowing that you have limited time, we highly appreciate your efforts.

After analyzing the results from the first and second Delphi round, we can conclude to have reached consensus on the relevance and feasibility to diagnose dehydration among nursing home residents within the nursing home, of 9 anamnestic methods, 8 physical symptoms and 3 blood tests. The specific items on which consensus was reached will first be presented to you in graphs. **This is just for your information; you don’t have to do anything with these results**.

The gold standard to confirm the diagnosis of dehydration, is blood testing. Before experts decide to carry out blood tests, there must be a suspicion of dehydration. The anamnesis and / or the presence of physical symptoms are of great importance to make experts suspect dehydration among nursing home residents. Therefore we have developed **a diagnostic strategy** to suspect and confirm the presence of dehydration among nursing home residents residing in the nursing home, based on the methods consensus was reached in terms of relevance and feasibility by you and 34 other experts who have participated in this Delphi study.

In this third Delphi round we kindly ask you **to indicate whether or not you agree with this step-by-step diagnostic strategy as a gold standard to diagnose dehydration in nursing home residents.**Whilst we recognise that blood tests are not possible in all nursing home settings, we would like you to consider what would be the gold standard diagnostic process if these tests were available to you. Please answer this question with ''yes'' or ''no''.

Filling in the questionnaire takes maximum 5 minutes.

**Anamnesis (medical history)**
Consensus (≥75%) was reached in the first and second Delphi round on the following anamnestic item to diagnose dehydration among nursing home residents residing in the nursing home in terms of **relevance and feasibility**


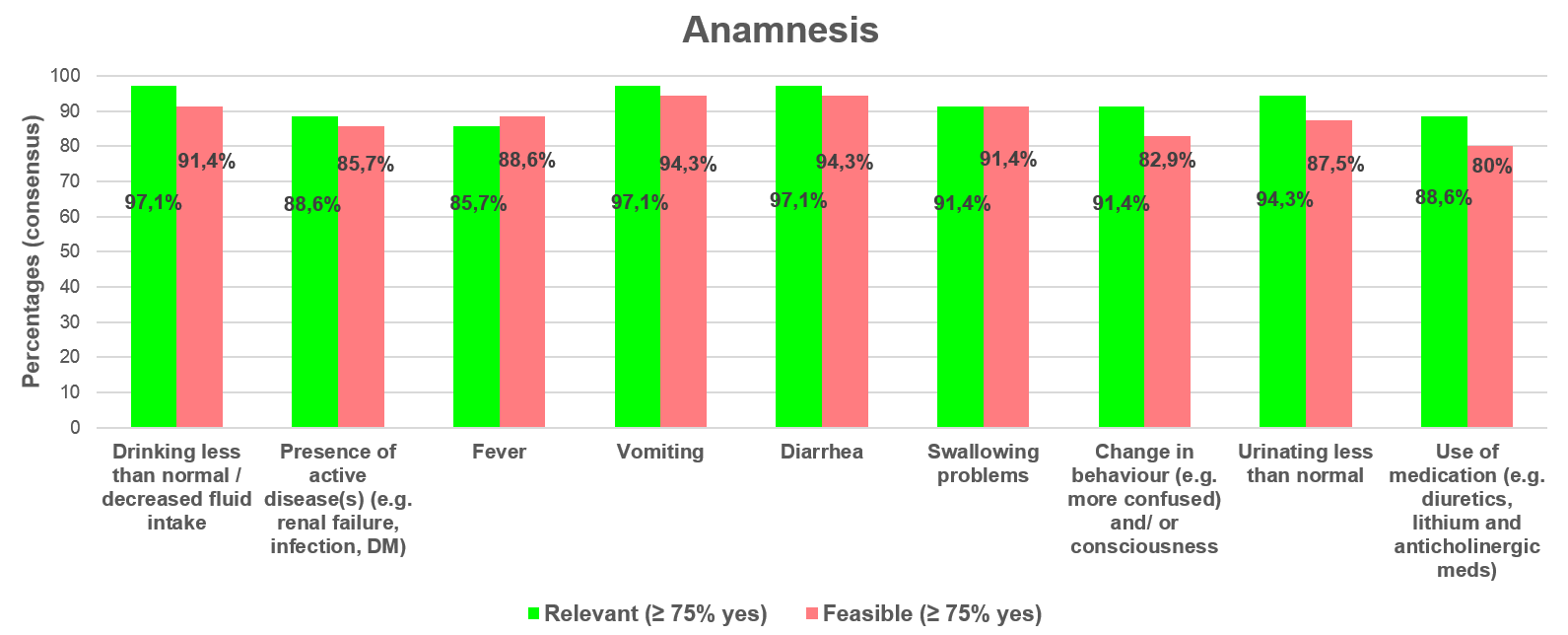


**Physical diagnosis**
Consensus (≥75%) was reached in the first and second Delphi round on the following physical symptoms to diagnose dehydration among nursing home residents residing in the nursing home in terms of **relevance and feasibility**


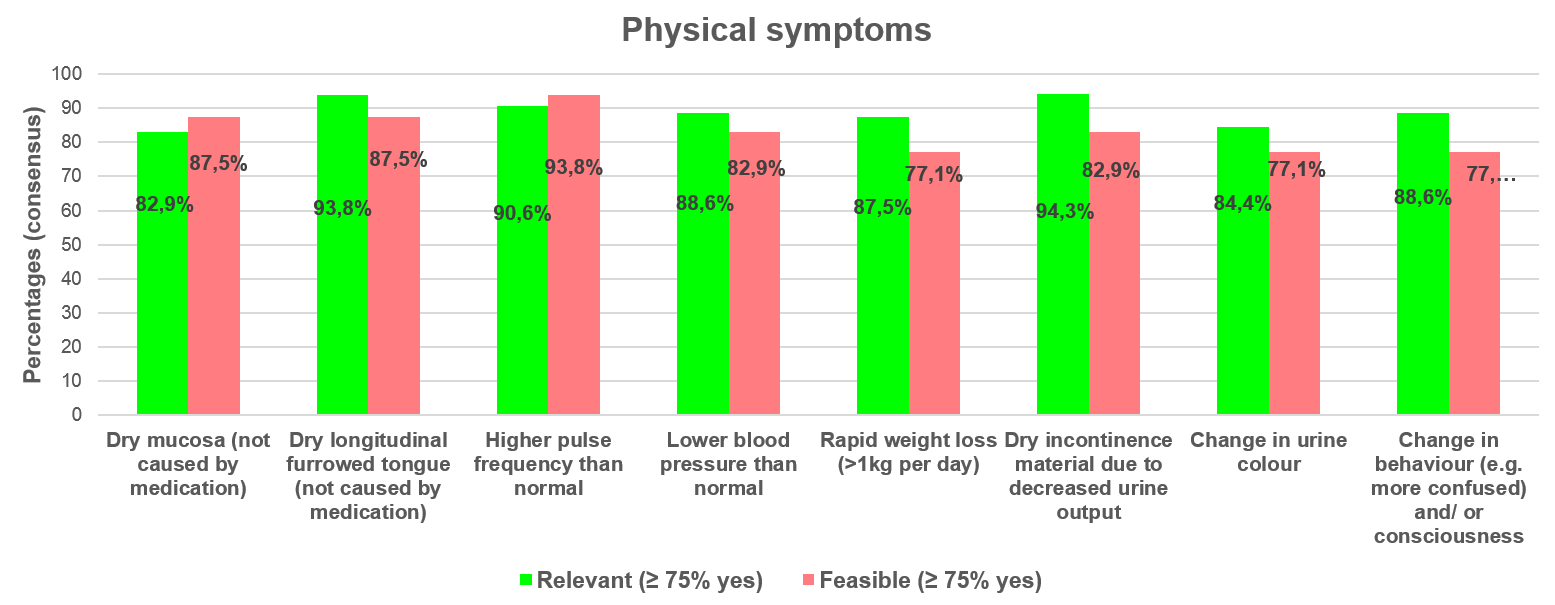


**Blood testing**
Consensus (≥75%) was reached in the first and second Delphi round on the following blood tests to diagnose dehydration among nursing home residents residing in the nursing home in terms of **relevance and feasibility**


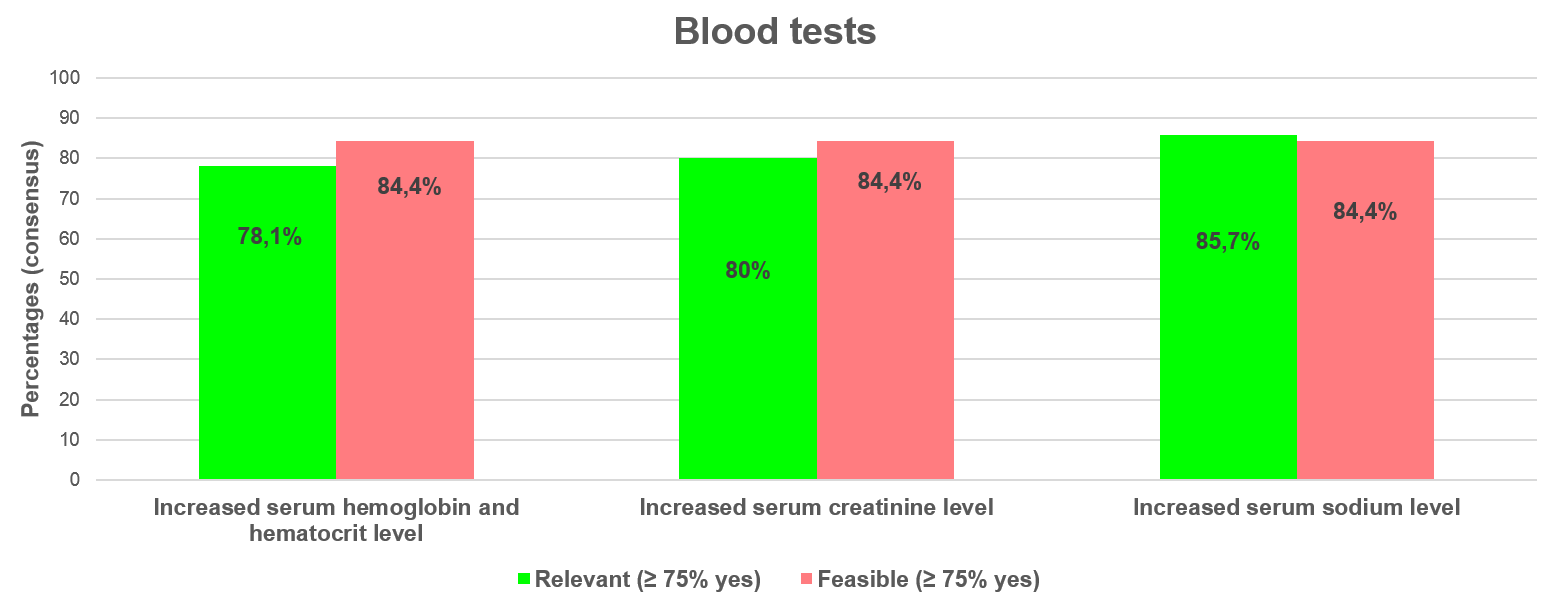


Q1 **Do you agree with this step-by-step diagnostic strategy as a gold standard to diagnose dehydration in nursing home residents? Whilst we recognise that blood tests are not possible in all nursing home settings, we would like you to consider what would be the gold standard diagnostic process if these tests were available to you.**


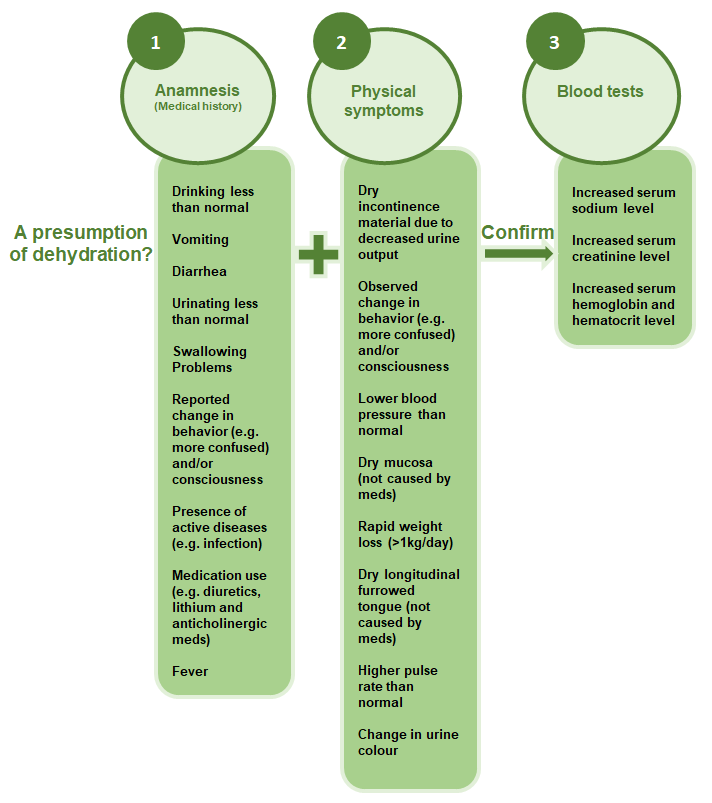


- **Yes**
- **No, because** ________________________________________________

**Thank you for your valuable cooperation in this Delphi round! Finish this questionnaire and record your answers by clicking on the ´next´ button.**
